# Antenatal care in rural Bangladesh: current state of costs, content and recommendations for effective service delivery

**DOI:** 10.1186/s12913-019-4696-7

**Published:** 2019-11-21

**Authors:** Youngji Jo, Kelsey Alland, Hasmot Ali, Sucheta Mehra, Amnesty E. LeFevre, Semee (Esther) Pak, Saijuddin Shaikh, Parul Christian, Alain B. Labrique

**Affiliations:** 1 Johns Hopkins Bloomberg School of Public Health, Johns Hopkins University, 615 N. Wolfe Street, Baltimore, MD 21205 USA; 2 JiVitA Program, Johns Hopkins University, Gaibandha, Rangpur, Bangladesh

**Keywords:** Antenatal care, Cost, Service delivery, Bangladesh

## Abstract

**Background:**

Measurement of antenatal care (ANC) service coverage is often limited to the number of contacts or type of providers, reflecting a gap in the assessment of quality as well as cost estimations and health impact. The study aims to determine service subcomponents and provider and patient costs of ANC services and compares them between community (i.e. satellite clinics) and facility care (i.e. primary and secondary health centers) settings in rural Bangladesh.

**Methods:**

Service contents and cost data were collected by one researcher and four interviewers in various community and facility health care settings in Gaibandha district between September and December 2016. We conducted structured interviews with organization managers, observational studies of ANC service provision (*n* = 70) for service contents and provider costs (service and drug costs) and exit interviews with pregnant women (*n* = 70) for patient costs (direct and indirect costs) in health clinics at community and facility levels. Fisher’s exact tests were used to determine any different patient characteristics between community and facility settings. ANC service contents were assessed by 63 subitems categorized into 11 groups and compared within and across community and facility settings. Provider and patient costs were collected in Bangladesh taka and analyzed as 2016 US Dollars (0.013 exchange rate).

**Results:**

We found generally similar provider and patient characteristics between the community and facility settings except in clients’ gestational age. High compliance (> 50%) of service subcomponents were observed in blood pressure monitoring, weight measurement, iron and folate supplementation given, and tetanus vaccine, while lower compliance of service subcomponents (< 50%) were observed in some physical examinations such as edema and ultrasonogram and routine tests such as blood test and urine test. Average unit costs of ANC service provision were about double at the facility level ($2.75) compared with community-based care ($1.62). ANC patient costs at facilities ($2.66) were about three times higher than in the community ($0.78).

**Conclusion:**

The study reveals a delay in pregnant women’s initial ANC care seeking, gaps in compliance of ANC subcomponents and difference of provider and patient costs between facility and community settings.

## Background

Antenatal care (ANC) is widely recognized as an accessible and cost-effective method to improve maternal and perinatal health outcomes [1]. It offers the opportunity to connect women to the health system, and improve maternal and child health outcomes through prevention, health promotion and treatment during pregnancy. ANC can increase access to and chances of using a skilled attendant at birth around labor and delivery – which is when most maternal and newborn deaths occur – through a birth and emergency preparedness plan [2]. Studies show that attending at least four quality ANC sessions is an effective strategy to increase skilled birth attendant use and institutional delivery [3, 4].

Despite substantial progress in primary health care over the last decades, only 21% of pregnant women in Bangladesh receive at least four ANC visits, just 31% of births are delivered at health facilities, and skilled birth attendants assist only 41% of women during childbirth in Bangladesh [5]. A lack of access to health providers and facilities has contributed to nearly three in four (73%) mothers in Bangladesh not receiving four or more ANC visits from skilled health professionals, let alone the eight ‘contacts’ recently recommended by the World Health Organization (WHO) [6]. Further, while 74% of urban women receive ANC from a trained provider, only 49% of rural women have such access [7]. Improving access to quality ANC and sustaining its implementation must be prioritized for the country to achieve the health Sustainable Development Goals.

Measurement of ANC service coverage is often limited to the number of contacts or type of providers, reflecting a gap in the assessment of quality as well as cost estimations and health impact; this is exacerbated by fragmented, pluralistic health systems and imprecise data on health system performance. This study sought to fill a part of this data gap and promotes current efforts to understand ‘effective’ coverage (i.e. proportion of the target population or population in need is actually benefiting from complete – effective – packages of interventions) beyond ‘contact’ coverage (i.e. proportion of the target population or population in need contacted by health service providers) of ANC services [8]. With a special focus on identifying gaps and barriers in effective service delivery, this analysis aims to describe the service subcomponents, costs and characteristics of patients and service provision among various community and facility settings in a rural setting of northern Bangladesh.

## Methods

### Study setting

The study was conducted between September and December 2016 at the JiVitA maternal and child health research site [9] in rural Gaibandha district in northwest Bangladesh, with an estimated 2.6 million population and 45,000 pregnant women each year. A previous study estimated that 18.5% of pregnant women reported receiving any one ANC service, of which a majority received were delivered by either a community-based nongovernmental organization (NGO), i.e. BRAC (71%), or government health workers (15%) [6].

### Community setting

Major stakeholders (in the formal sector) of community-based services in Gaibandha district include the government and the country’s largest NGO, BRAC, and the Smiling Sun franchise satellite clinics. ANC services at the community level are provided mainly by community health workers (CHWs) via household visits anchored to mobile or temporal clinics such as satellite clinics, which are often set up on certain days in a community member’s home or any public gathering space. For BRAC, a CHW’s working areas are divided into three to five clusters and each cluster generally consists of 75 to 100 households. In each cluster, a satellite clinic is set up twice a month. Community mobilizers, who visit each house for pregnancy surveillance, family planning and other health promotion efforts in their assigned catchment areas, inform the pregnant women of the specific ANC provision dates and satellite clinic location in advance of the ANC visits. There are in total 638 estimated satellite clinics and more than 3000 CHWs – government employed CHWs including 421 Family Welfare Assistants (FWAs), 72 Family Welfare Visitors (FWVs), and 53 Family Planning Inspectors (FPIs) and BRAC employed CHWs including 2000 Shyastho Kormis (SK) and 200 Shyastho Shebikas (SS) – who conduct pregnancy surveillance of 560,000 households of women of reproductive age in Gaibandha district (2.6 million population). There are several informal providers (e.g. traditional healers, village doctors) in the community setting but we did not include this group in our study.

### Facility setting

ANC service provision at the facility level involves different levels of public actors, NGOs, and private actors throughout primary and secondary care. Public facilities include Community Clinics (CC), Family Welfare Centers (FWC), Union-sub centers, Upazila Health Complex (UHC), and Maternal and Child Welfare Center (MCWC). Other facilities include the Smiling Sun franchise static clinics and private emergency obstetric care (EMoC) clinics. ANC services at the facility level are delivered by a range of service providers from FWVs, paramedics, Sub-Assistant Community Medical Officer (SACMO), to MBBS (i.e. degree in medicine) doctors. Due to the shortage of skilled health providers, ANC services are provided only on certain days of the week in a clinic (e.g. every Saturday or Tuesday; days vary in facilities), as one provider has to cover multiple facilities in a broad catchment area. CHWs inform pregnant women of these dates during routine household visits or at community satellite clinics. There are a total of 299 CCs, 54 FWCs, six UHCs, and two MCWCs; there are four Smiling Sun clinics and no BRAC facilities in Gaibandha district. There are several private clinics and pharmacies in the facility setting but we did not include these groups in our study.

### Data collection

**Fig. 1:**
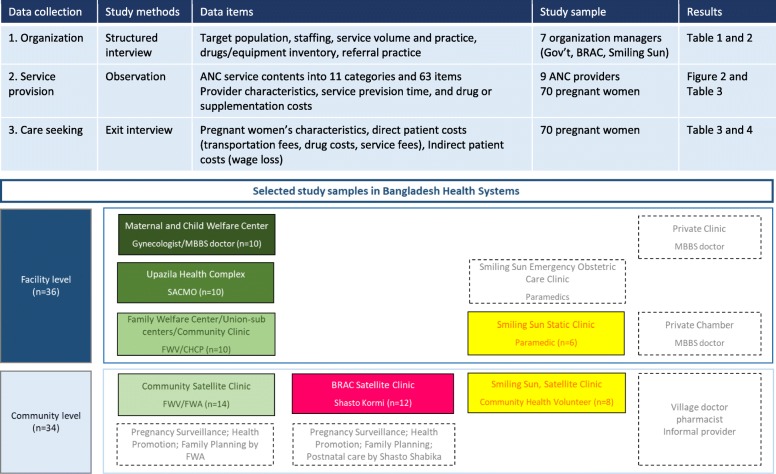
Analytic approach and selected study samples in Bangladesh health systems

Adapting standardized service delivery and quality assessment guidelines such as the WHO’s Service Availability and Readiness Assessment (SARA), the World Bank Group’s Quantitative Service Delivery Surveys (QSDS) and USAID’s Service Provision Assessment (SPA) [10–12], we devised service costing and coverage tools that can account for various stakeholders at different levels of care (e.g. community and facility settings) to capture service practice and commodities such as equipment (e.g. blood pressure meter) and supplements (e.g. micronutrient or iron-folic acid tablet). The tool collected a total of 63 subservice content items grouped into 11 general categories, which are listed in Fig. 1. Provider cost includes service costs (based on provider’s service time) and drug costs. Patient cost data includes indirect costs (based on estimated wage loss to seek care) and direct costs (based on any medical or other expenses to seek care such as transportation fees or food costs). Data collection was carried out by one researcher and four interviewers. The provider’s service provision observation and client exit interviews were conducted by two interviewers as a team for each task simultaneously so observation and exit interview data could be matched with same individual patient identification numbers.

The study uses three data collection modules (Fig. 1). The first is a structured interview guideline, which an interviewer asks organization managers and health service providers for information on overall organization governance, staffing structure, service capacity/volume, referral practice, drug or equipment inventories and other related issues. We interviewed each of the organization representatives (Deputy Director of Family Planning, BRAC district manager and Smiling Sun USAID-DFID NGO Health Service Delivery Project country representative) and clinic managers (MCWC, UHC, FWC, CC, and Smiling Sun). The second module constitutes observations of actual service provision, which consists of 63 ANC service subcomponent items, grouped into 11 categories, in a broad spectrum of clinical history, general examination, counseling, screening and lab testing and treatment. It also asks about the type of providers, and measures service provision time per patient and any consumed commodities during specific service provision activities. The third module comprises exit interviews with clients who received the service. The interviews occurred at the end of each service provision observation, and the interviewer inquired about direct costs such as service fees, transportation fees and drug costs, as well as any indirect costs such as loss of wages due to the care seeking. Wage loss was calculated by total time spent to seek care multiplied by estimated daily income based on the reported clients’ average monthly salary. Similarly, service provision cost was calculated by average service time per patient multiplied by estimated daily income estimated from the providers’ monthly salary.

### Sample recruitment

The choice of facilities or communities was decided through purposive sampling on the basis of facilities’ service schedule, volume of service provision, and operational settings, which can be generally be perceived as representative of the organizations’ routine practices by local experts. At each respective service provision site, an organization manager was recruited for structured interviews, as were one to two generally representative ANC service providers for an observation study. The identification of pregnant women and providers within a community or facility was purposively made on the scheduled days of observation at satellite clinics, CCs, FWCs, UHCs, MCWCs and Smiling Sun static clinics. At the scheduled community and facility sites, pregnant women were approached by research staff in the ANC waiting area and requested to provide consent for both observation and exit interviews prior to the start of ANC clinical services.

### Sample size

In total, we observed 70 ANC service provisions by nine providers (four providers in community settings and five in facility settings) and conducted exit interviews with 70 pregnant women (34 in community settings and 36 in facility settings) for the patient cost survey. The client sample includes service provision by FWVs (14 samples), BRAC’s SKs (12 samples), and Smiling Sun Paramedic and counselors (8 samples) in community settings and service provision by FWVs in FWCs and CCs (10 samples), SACMOs in UHCs (10 samples), an MBBS/Gynecologist doctor in MCWC (10 samples) and paramedics in the Smiling Sun static clinic (6 samples).

### Data analyses

First, service provision characteristics were described in the aspects of general scope of catchment area, service contents, staffing and general drug and supplementary items used for ANC based on the structured interview. Characteristics of the study samples including both providers and pregnant women were described overall and independently for each group. For the comparison of the mothers’ characteristics, we used Fisher’s exact tests to assess difference in patients’ characteristics between the community and facility groups. Second, we analyzed ANC observation data and described ANC service subcontents, including counseling and clinical care provided, at the community and facility levels, independently and overall. Given that the relevant ANC service components are likely to differ by pregnancy stages, compliance percentage of each service item was calculated based on the number of pregnant women in the relevant gestational age as a denominator (Additional file 1: Table S1). Based on the finding, we assessed gaps and difference in the content of care provided within and between groups. Data about service practice from the observations were also used to contextualize and validate the findings from the in-depth interviews.

Lastly, we estimated the cost of ANC service provision including service costs and drug costs. Service costs are calculated based on staff category, average monthly salary and average service time per patient. Drug costs are calculated based on unit price and quantity of commodities provided (e.g. iron and folic acid or calcium). We did not include infrastructure, capital equipment or overhead costs in calculating service provision costs. We estimated the cost for patients seeking ANC including direct and indirect costs. Direct patient costs are calculated based on any out-of-pocket payments for services or drugs and transportation. Indirect patient costs are estimated as amount of wage loss based on total time of care seeking and the households’ occupation and monthly salaries (Table 1). To avoid bias from extreme values in our survey data, we estimated median values based on an interquartile range of 25% and 75% of the dataset excluding missing values.

**Table 1:** Provider and patient costs (in 2016 US Dollars) for antenatal care in Gaibandha district

Level of care (*n* = number of pregnant women)			Community Level (*n* = 34)			Facility Level (*n* = 36)			
Care setting			Gov’t (*n*=14)	BRAC (*n*=12)	Smiling Sun (*n*=8)	Community Clinic (*n*=10)	Upazila Health Complex (*n*=10)	Maternal and Child Welfare Center (*n*=10)	Smiling Sun (*n*=6)
Provider costs	Service costs	Staff level	Family Welfare Visitor	Shyastho Kormi	Paramedic	Family Welfare Visitor	Nurse	Family Welfare Visitor	Paramedic & Counselor
		Staff monthly salary ($)	182-390	78	286	182-390	208-455	182-390	286
		Service time per patient (min)	6 (5-10)	25 (21-25)	10 (9-10)	5 (5-6)	17 (10-20)	10 (5-14)	28 (18-30)
		Total service costs	0.17 (6%)	0.18 (21%)	0.27 (28%)	0.13 (4%)	0.55 (16%)	0.27 (9%)	0.75 (51%)
	Drug costs^a^	Iron & Folic acid ($)	0.32-0.39	0.13	0.13	0.32-0.39	0.32-0.39	0.32-0.39	0.13
		Calcium ($)	0.45-0.65	0.13-0.19	0.13	0.45-0.65	0.45-0.65	0.45-0.65	0.13
		Vitamin B Complex ($)	n/a	0.26-0.47	0.45	n/a	n/a	n/a	0.45
		Misoprostol ($)	1.95	n/a	n/a	1.95	1.95	1.95	n/a
		Total drug costs	2.86 (94%)	0.66 (79%)	0.71 (72%)	2.86 (96%)	2.86 (84%)	2.86 (91%)	0.71 (49%)
Total provider costs			3.03	0.84	0.98	2.99	3.41	3.13	1.46
Average provider costs (USD, 2016)			$1.62			$2.75			
Patient costs^b^	Indirect costs	Hourly wage^c^ ($)	0.58 (0.36-0.71)	0.52 (0.43-0.69)	0.88 (0.42-0.10)	0.48 (0.36-0.92)	0.52 (0.44-0.84)	0.62 (0.44-0.84)	0.66 (0.36-0.66)
		Travel time (a round trip, min)	20 (10-55)	13(6-33)	10 (10-17)	10 (10-20)	48 (41-60)	120 (70-120)	40 (25-70)
		Waiting time (min)	13 (5-56)	15 (2-30)	6 (4-17)	20 (8-30)	40 (15-105)	120 (90-210)	8 (1-10)
		Consultation time (min)	10 (8-10)	30 (24-35)	18 (9-20)	4 (2-9)	13 (10-28)	10 (10-20)	30 (23-41)
		Pharmacy time (min)	0 (0-0)	0 (0-1)	0 (0-0)	0 (0-0)	1 (0-4)	0 (0-0)	0 (0-0)
		Total time (min)	73 (46-114)	78 (43-97)	34 (27-44)	43 (27-52)	153 (78-182)	260 (195~292)	90 (70-106)
		Total wage loss (total time x hourly wage)	0.70 (0.48-0.96) (100%)	0.49 (0.31-1.03) (43%)	0.49 (0.31-0.91) (100%)	0.44 (0.25-0.61) (77%)	1.19 (0.69-2.29) (80%)	2.25 (1.40-5.96) (42%)	0.81 (0.40-0.94) (53%)
	Direct costs	Admission fee ($)	0	0	0	0	0.04 (0.04-0.04)	0	0
		Medical service fee ($)	0	0.65 (0.65-0.65)	0	0	0	1.89 (1.20-2.93)	1.56 (1.02-1.62)
		Transportation ($)	0	0	0	0.13 (0-0.2)	0.45 (0.32-0.56)	0.52 (0.23-0.91)	0.65 (0.52-0.78)
		Others ($)	0	0	0	0	0	0.71 (0.13-1.30)	0
		Total direct costs	0 (0%)	0.65 (0.65-0.65) (57%)	0 (0%)	0.13 (0-0.2) (23%)	0.49 (0.04-0.56) (20%)	3.12 (1.27-4.48) (58%)	2.21 (0.59-1.36) (47%)
Total patient costs			0.70	1.14	0.49	0.57	1.68	5.37	3.02
Average patient costs (USD, 2016)			$0.78			$2.66			
Average provider and patient costs (USD, 2016)			$2.40			$5.41			

^a^Providers’ monthly salary estimates and drug costs are based on interviews with organization managers and drug inventory documents in the facilities. Provision of medicines and supplements may vary depending on government supply in stock. Unit drug cost are slightly different by product brands

^b^Patient costs are based on direct service observation (n=70) and exit interviews with pregnant women (*n* = 70) Wage, time, and patient costs data represent median estimates and interquartile ranges (25% and 75%)

^c^Hour wages are estimated based on women/husbands’ reported monthly salaries. Total wage loss was calculated based on multiplying the estimated hourly wage and total time spend to seek care

### Ethical statement

The study received ethics approval from the Bangladesh Medical Research Council (Reference number: BMRC/NREC/2013–2016/375, dated 14/10/2015) and the Johns Hopkins Bloomberg School of Public Health Institutional Review Board (IRB00006999). For the data from the observation study and exit interviews, subjects enrolled in the study completed written consent procedures from all participants.

## Results

### Service provision

Table 2 summarizes the characteristics of service provision in community settings. In general, one FWA covers 800 to 1000 households and one NGO CHW covers 350 to 450 households for pregnancy surveillance and family planning and identifies six to nine pregnancies per month in her catchment area. One CHW visits 15 to 30 households for routine surveillance per day, and an additional 20 to 30 min per household for travel and service time. The range of family planning instruments, drugs and supplies varies across satellite clinics. Most basic ANC services – clinical history, physical examination, consultation, simple screening or lab test services (e.g. hemoglobin and urine test) and supplementation (e.g. iron and folate acid provision) – were provided for free at the community level in government satellite clinics by FWVs and at a minimal fixed cost such as 20 taka (US$0.24) by Smiling Sun clinics. Most commonly available supplies across the agencies were iron and folic acid, misoprostol and paracetamol.

**Table 2:** Characteristics of antenatal care service provision in community settings in Gaibandha district

Type of setting	Community Level (Satellite clinics)		
	Government Family Welfare Assistant/Visitors (FWA, FWV)	BRAC *Shyastho Kormis (SK)*, *Shyastho Shebikas (SS)*	Smiling Sun Community Service Promotors (CSP)
Service capacity for target population	- 1 FWA covers 1 ward (population 5000-6000) and is responsible for 800-1000 HHs (eligible couple with reproductive age women) for pregnancy surveillance/family planning; 1 FWA/FWV identifies about 6-7 pregnancies per month in her catchment area. - 1 FWA visits about 30 HHs for routine surveillance per day in her catchment area - 60-70 pregnant women per month were reported in 1 union	-1 *Shyastho Kormis (SK)* covers 360 HH for pregnancy surveillance/family planning ; 1 SK identifies about 9 pregnancies a month in her catchment area -1 SK visits about 15 HHs for routine surveillance activities per day in her catchment area.	-1 Community Service Promoters (CSP) covers 421-430 HH for pregnancy surveillance/family planning; 1 CSP identifies about 9 pregnancies a month in her catchment area -1 CSP visits about 20 HH for routine surveillance activities per day in her catchment area
Service provision and costs	-Clinical history, Examination, Counseling -All free	-Clinical history, Examination, Counseling -Lab service (hemoglobin, Urine tests) -Treatment (iron and folate) ($0.6/50 taka)	-Clinical history, Examination, Counseling ($0.24/20 taka)
Staffing	-In Gaibandha district (Population: 2.6 million) there are family planning inspectors (53), *Sub-Assistant Community Medical Officers (SACMO) (*49), Family welfare visitors (FWV) (72), Family welfare assistants (FWA) (421), Community health care providers (291) -1 union (population 18,000) has 3 community clinics and 8 satellite clinics including 1 SACMO, 1 FPI, 1 FWV, 5 FWAs	-1 upazila (10~15 unions) has 1 manager, 10 program officers and 35 *Shyastho Kormis (SK)*, and 399 *Shyastho Shebikas (SS)* -BRAC only provides satellite clinics by SK/SS and there are no static clinics in the Gaibandha district.	-In Gaibandha, there are total 36 satellite clinics in 3 unions. 1 union has 18 satellite clinics including 1 Paramedics, 18 CSPs. The paramdics are service providers, CSPs provide surveillance, family planning, community mobilization support.
Drugs and supplies	-Family planning (male condoms, oral contraceptive pills, intrauterine devices (IUDs), implants, injectable contraception) which are all free. -Maternal care (iron tablets, folic acid, misoprostol tablet, oxytocin injection, paracetamol)	-Family planning (male condom, oral contraceptive pill which are free cost) -Maternal care (iron and folic acid, misoprostol, paracetamol, oral rehydration)	-Family planning (male condom, oral contraceptive pill, emergent contraceptive pills, IUDs, implants, injectable contraception, sterilization surgery which are free from government support) -Maternal care (iron and folic acid, misoprostol, Azithromycin, Cefixime, Betamethasone, Dexamethasone, Nifedipine, methyldopa)
Referral practice	Referral was most frequent during labor and delivery. During the pregnancy period, ANC visits during the first (8-12 weeks base on the guideline but actual first visit timing of some pregnant women may be beyond 12 weeks) and fourth (36-38 weeks). Reasons for referral are varied (e.g. vaginal bleeding, fever, headache or blurred vision, cough or difficulty breathing, fetal movement, convulsion, high blood pressure). Referrals are often made by verbally advising the client to be hospitalized urgently, explaining the reason for referral, occasionally giving a referral slip to the caretaker, or explaining where and when to go with a health professional’s contact information.		

Table 3 summarizes the characteristics of service provision in facility settings. The organizational and staffing structures vary by agency; in the Gaibandha district, three community clinics and eight satellite clinics exist in one union (which is the smallest rural administrative unit: approximate population size is 18,000), consisting of one SACMO, one FPI, one FWV and five FWAs. In the Smiling Sun clinic, one paramedic and 18 Community Service Promoters work for 18 satellite clinics in one union. Service components and levels of qualified health providers were incrementally more available from primary to tertiary care settings. While most services were free or of minimal costs at public clinics, drugs were often not sufficient for the needs and not procured in a timely manner, and patients had to purchase them at pharmacies outside of the facilities. Services such as screening, lab tests, supplementation or treatments were available for free in government facilities and for a wide range of user fees at the Smiling Sun static clinic (e.g. hemoglobin/proteinuria, $0.36/30 taka; urine for routine examination, $1.19/100 taka; blood grouping, $0.24/20 taka; syphilis, $1.19/100 taka).

**Table 3:** Characteristics of antenatal care service provision in facility settings in Gaibandha district

Type of setting	Facility Level (Primary / secondary healthcare centers)			
	Family Welfare Centers / Community Clinic	Upazila Health Complex	Maternal and Child Welfare Center	Smiling Sun
Number of the facilities	40-50 clinics	6 clinics (31 beds per clinic)	1 clinic (20 beds per clinic)	4 clinics (2 clinics are emergency obstetric centers and 2 clinics are static clinics with no child delivery service)
Service provision and costs	-Clinical history, Examination, Counseling -Supplementation and treatment (bacteriuria, TT, iron and folate) -All services are free	-Clinical history, Examination, Counseling -Screening/lab test (hemoglobin, proteinuria, urine, blood group, ultrasonogram, syphillis) -Supplementation and treatment (syphillis, bacteriuria, TT, iron and folate, calcium, balanced energy supplementation, micronutrient, MgSO4, hypertentive disease, diabetes screening, malaria case managmenet) -$0.036/3 taka for outpatient care; $0.06/5 taka for inpatient care	-Clinical history, Examination, Counseling -Screening/lab test (hemoglobin, proteinuria, urine, blood group, ultrasonogram, syphillis) -Supplementation and treatment (syphilis, bacteriuria, TT, iron and folate, calcium, balanced energy supplementation, micronutrient, MgSO4, hypertentive disease, diabetes screening, malaria case managmenet) -All services are free	-Clinical history, Examination; Counseling -Screening/lab test (e.g. hemoglobin/proteinuria, $0.36/30 taka; Urine for routine examination, $1.19/100 taka; Blood grouping, $0.24/20 taka; syphilis $1.19/100 taka) -Supplementation and treatment (TT, iron and folate) -Different prices per client’s economic status ($0.24/20 taka, $0.12/10 taka, free)
Staffing	Nursing professional /FWV, Paramedics, FWA, Cleaner, Guard	Doctor, Family planning officer, Non-physician, Nursing professional, Paramedics, Pharmacy technologist, MIS manager, Messenger/ driver	General doctor, Non-physician clinician, Anesthetist, Nursing professional, Driver, Cleaner, Guard,	General doctor, Paramedics, Counselor, Lab technician, MIS manager, Community health volunteers, Messenger, Cleaner, Guard
Drugs and supplies	-Maternal care (iron, folic acid, misoprostol, oxytocin, paracetamol)	-Family planning (male condom, oral contraceptive pills, IUDs, implants, injectable, sterilization) -Maternal care (iron, folic, TT, paracetamol, ORS)	-Family planning (male condom) -Maternal care (iron, folic acid, TT, sodium chloride, Calcium gluconate, ampicillin powder, hydralazine, azithromycin cap, Cefixime cap, Benzathine benzylpenicillin, Nifedipine, Methyldopa, ORS)	-Family planning (male condom, oral contraceptive pill, IUD, injectable) -Maternal care (iron, folic acid, TT, sodium chloride injectable, magnesium, ampicillin powder, gentamicin injection, hydralazine, metronidazole injection misoprostol, azithromycin, cefixime, benzathine benzylpenicillin, oxytocin, paracetamol, oral rehydration)
Referral practice	Referral was made from primary care centers/clinics toward secondary and tertiary level hospitals. (FWC→UHC→MCWC). For certain complicated obstetric deliveries (e.g. C-sections), some patients sought care at private clinics that mainly operated on child delivery in this setting. There were limited public ambulances to support patients during labor and child delivery but there were some community based social networks that support vehicles or mobilize funding for poor households.			

### Referral practices

The interviews with providers revealed that ANC visits during the first visit (8 to 12 weeks based on the guideline, but the actual timing of first visit for some pregnant women may be beyond 12 weeks) and fourth visit (36 to 38 weeks) were the most frequent, with labor and delivery triggering the most frequent referrals. Reasons for referral varied (e.g. vaginal bleeding, fever, headache or blurred vision, cough or difficulty breathing, fetal movement, convulsion, high blood pressure). Referrals were often made by verbally advising the client to be hospitalized urgently, explaining the reason for referral, occasionally giving a referral slip to the caretaker, or explaining where and when to go with a health professional’s contact information. However, there is no systematic mechanism to ensure that a patient has acted on a referral, visited a clinic and received appropriate care.

### Care seeking

While we found generally similar provider and client characteristics (Table 4) between the community and facility levels, there was a statistically significant difference in the clients’ gestational age (GA). A greater proportion of women in earlier pregnancy (GA less than 12 weeks) sought care at community level than at facilities, while women in later pregnancy stages (GA between 13 and 26 weeks) tended to seek care at the facility level. However, there was no substantial difference (for GA between 27 and 32 weeks) or only small samples observed (for GA between 33 and 38 weeks) between community and facility levels. Further, women’s first ANC visit to either a community or a facility was sought at a median of GA 20 weeks (a range between 16 and 28 weeks), which is far later than the current guideline of no later than 12 weeks. In other words, out of 36 women who received their first ANC visit, only seven (20%) women’s GA was within 12 weeks.

**Table 4:** Study population characteristics in Gaibandha district

Service provision characteristics		Community (*n* = 4)		Facility (*n* = 5)		Fisher's exact test
Provider	Employee designation	Family Welfare Visitor, Paramedic BRAC Shyastho Kormi		Family Welfare Visitor, Paramedics, Nurse, Doctors		
	Age (years)	26, 28, 35, 36 years old		26, 29, 40 years old		
	Schooling (years)	11-14 years		10-14 years		
	Working on ANC service (years)	3-10 years		3-10 years		
	Last training received (years)	1-10 years ago		3-10 years ago		
Mother's characteristics		Community (*n* = 34)		Facility (*n* = 36)		*P*-value
		n	%	n	%	
Age	<20	13	38%	9	25%	0.66
	20-34	20	59%	26	72%	
	35-49	1	3%	1	3%	
Parity	First pregnancy	18	53%	13	36%	0.23
	Not first pregnancy	16	47%	23	64%	
GA	Within 12 weeks	8	24%	2	6%	0.01
	13-26 week	6	18%	19	53%	
	27-32 week	14	41%	13	36%	
	33-38 week	6	18%	2	6%	
ANC	1st visit	14	41%	22	61%	0.30
	2nd visit	9	26%	6	17%	
	3rd visit	8	24%	4	11%	
	> 4th visits	3	9%	4	11%	
Literacy	Yes	32	94%	30	83%	0.26
	No	2	6%	6	17%	
Schooling	No schooling	5	15%	5	14%	0.08
	Class 1~9 completed	25	74%	21	58%	
	Secondary/High school completed	3	9%	7	19%	
	Degree or higher	1	3%	3	8%	
Household occupation	Informal (own farm/unskilled labor/own business)	28	82%	27	75%	0.24
	Formal (private service/government)	6	18%	9	25%	

### Service contents

**Fig. 2:**
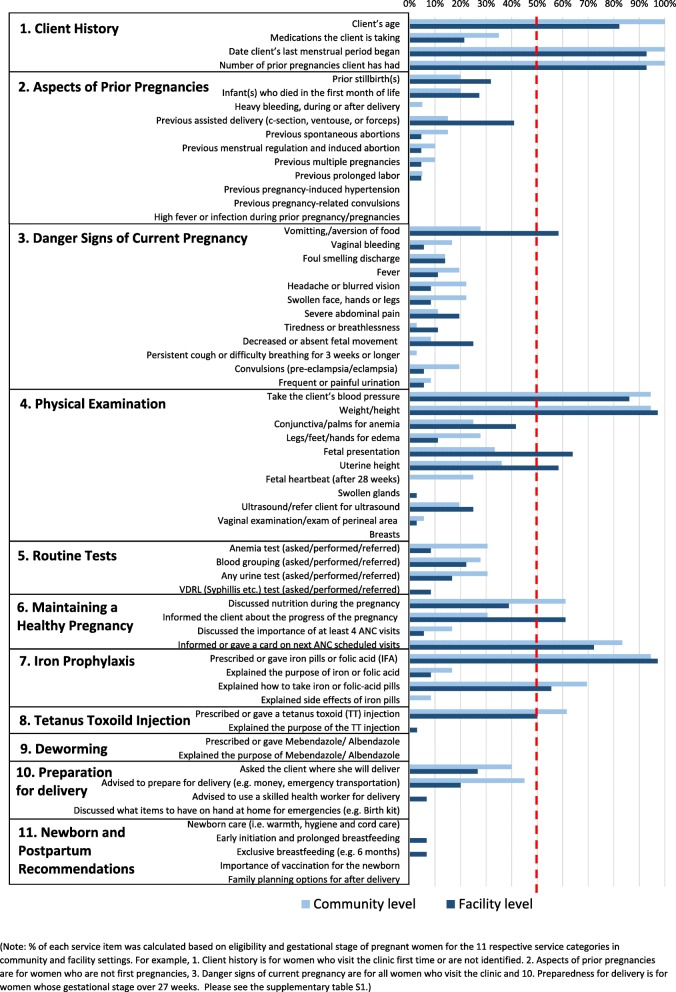
Percentage distribution of antenatal care service contents in community and facility levels

As expected, community-level ANC visits were rudimentary in nature, covering counseling and certain basic screening and preventive measures, while more specialized laboratory tests and treatments were only available at the facility level. Figure 2 shows that out of the 11 service categories, service contents or provision are lacking in the areas of prior pregnancies, danger signs of current pregnancy, routine tests, deworming, preparation for delivery, and newborn and postpartum recommendations in both community and facility settings. Out of 63 subservice contents items, only 10 (17%) and 12 (20%) items show relatively high (> 50%) compliance coverage in community and facility settings. These include blood pressure monitoring, weight measurement, iron and folate supplementation given, and tetanus vaccine, while compliance was low in explaining the purposes or side effects of these services. Low compliance of service components (< 50%) was observed in physical examination such as edema and ultrasonogram, routine tests such as blood grouping and urine test and advice on high risk pregnancy in both community and facility settings.

### Costs

Table 1 shows that total cost (including both provider and patient costs) per ANC was estimated as $5.41 at facility level, which is about two times that at community level ($2.40). First, ANC service provision unit cost at the facility level ($2.75) was almost double that at the community level ($1.62). ANC patient costs at facility level ($2.66) were about three times higher than at community level ($0.78). Drugs, supplies, and diagnostic tests account for the largest share of provider unit costs for ANC in general, while indirect costs (wage loss) account for a greater share of patient unit costs than direct costs, except at the tertiary level where medical service fees represent the largest share. Within the facility level, due to the high volume of clients seeking care at government clinics, unit service provision time tends to be shorter at government clinics (e.g. 5 to 14 min per patient at MCWC) than NGO clinics (e.g. 18 to 30 min per patient at Smiling Sun clinic). Patients’ travel and waiting times were however considerable (e.g. 90 to 210 min at MCWC) when seeking care especially at secondary clinics at the facility level, which contributes to increase in patient cost.

## Discussion

The study describes the general characteristics of service provision and care seeking and reveals a delay in pregnant women’s initial ANC care seeking, gaps in compliance of ANC subcomponents and difference of provider and patient costs between facility and community settings in rural Bangladesh. While ANC consists of several subcomponents of services varying with pregnancy stages, major service content and compliance gaps were identified in the areas of some physical examinations (e.g. edema or ultrasonogram), routine tests (e.g. blood grouping or urine test), and counseling on high risk pregnancy such as prior pregnancy history, danger signs of current pregnancy and preparation for delivery. The study found that a greater proportion of women at GA 12 to 36 weeks sought care at the facility settings compared to community settings. However, the first ANC visit was substantially delayed, from 13 weeks up to 32 weeks.

Our findings are consistent with many recent studies in Bangladesh that the ANC service providers and the place of ANC visits as strong significant predictors of receipt of elements of ANC services [13]. A study showed that the odds of receiving the items of ANC contents was three times higher for mothers who attended skilled providers of ANC services (i.e. qualified doctor, nurse/paramedic, FWV), compared with the unskilled providers (FWA and community health care provider) [13]. In terms of service contents, other studies [13–16], including a study based on a large and nationally representative dataset (i.e. Bangladesh Health and Demographic Survey 2014) [13, 17] show similar trends and gaps, as blood pressure and weight measurement and abdominal examination are the most common ANC contents delivered, while counseling on danger signs, urine testing, blood screening and ultrasound were conducted less than half of the time during the ANC contacts. In terms of care seeking, the study also showed pregnant women’s timing of the first ANC contacts were delayed (i.e. only 11% initiating ANC in the first trimester of pregnancy) [13].

In terms of cost, while not much evidence exists, our study finding is similar to previous studies in Bangladesh revealed costs of delivering ANC services varied widely by type and location of clinics in Bangladesh; A study reported the average economic cost per ANC visit is $1.1 (79.2 taka) from BRAC’s Integrated Maternal Newborn Care Service program [18], while another study estimated average cost per antenatal care was ($7.03) from NGO care settings under Smiling Sun network in Bangladesh [19]. Another study on costing of maternal health services in Bangladesh suggests that a package of four ANC visits varies across type of facility, increasing from $1.5 (112 taka) in community clinics to $8.7 (673 taka) in district hospitals from a provider perspective [20]. On the other hand, household costs of three ANC visits with a midwife including transportation and care costs were estimated at about $1.7 (129 taka, adjusted to 2016 BDT) in Matlab [21]. These reflect the dramatically different level of permutations in diagnostic, care options available, a high variability of service volume as well as care-seeking patterns.

Given the nature of ANC service provision (which requires different combinations of counseling, examination, supplementation and treatment throughout pregnancy), measuring accurate coverage and cost is a complex task particularly in highly fragmented and pluralistic health systems, as is common in resource-limited settings. Considering other possible approaches (health information systems, household surveys, facility and provider surveys) [14, 22, 23], our study design was optimized to assess ANC service contents and costs. It was conducted in an efficient manner to capture high quality comprehensive data – objective and complete with low recall bias – based on direct performance observation with exit interviews (rather than self-report or administrative record) conducted simultaneously at the point of service received (instead of two independent data sources). Accordingly, it uniquely allowed us to understand when, how and what ANC services were delivered from and to whom at what costs. Further, the scope of sampling allowed for a consistent approach to examine comprehensive service provision channels for ANC services and to evaluate quality dimensions among various types and levels of facilities.

By focusing on the specific service provision points, the study evaluated where gaps exist in achieving effective coverage based on the local demand and supply characteristics. While the latest WHO guidelines for a positive pregnancy experience suggests a minimum of eight contacts to improve the utilization and quality of ANC [24], our study findings identify a number of critical health systems constraints which affect the quality of service and care-seeking behaviors to achieve this goal. We found that qualified providers are not enough, so one provider had to cover multiple clinics and catchment areas. Advanced diagnostic equipment, such as the ultrasonogram, was not available in community clinics, which need effective referrals to the clinic for those identified with danger signs. At facilities, drugs were not always constantly available, which requires additional out-of-pocket payment for patients for necessary treatment. Some women (12 out of 36) made their first ANC visits as late as 27 to 32 weeks of gestation, a major deviation from the standard guideline. This likely reflects both the inability of health workers to proactively visit all eligible pregnant women and promote timely care as well as demand-side barriers to care seeking, from cost and social boundaries to a lack of perceived need for ANC [14].

Given the barriers to service provision – including shortages of a qualified workforce, quality assurance mechanisms or incentive systems to promote quality care to clients – it is not surprising to find that providers cannot spend enough time to document in detail prior pregnancy histories and explain all the danger signs to a patient while many other patients are waiting to be seen in a crowded clinic. Similarly, in addition to the limited quality of the service provision side, it may be difficult to expect pregnant women to seek preventative ANC in a timely and frequently manner given the long travel distance, waiting time, and relatively high patient costs ($2) considering most households’ daily wage is $4-7. In facility care, the average patient cost per ANC ($2.66) comprises almost 50% of total societal cost per ANC ($5.41) with high out-of-pocket payments, which may hinder the achievement of effective and equitable ANC coverage for poor households.

## Recommendations

Given the delay in care seeking of the first ANC visit and coverage gap of service subcomponents, it is important to specialize and coordinate the roles of community and facility care in resource limited setting to improve effective service delivery. First, it is important to promote the capacity of community-level workers in identifying pregnant women and encouraging them to seek their first ANC at earlier gestational ages. Examination and identification of risk factors from danger signs and prior and current pregnancy can be more systematic and used to triage patients and prioritize service provision, acknowledging the human resource gap and targeting limited care provision capacity to patients who might benefit the most from ANC. Given the differing resource availability and capacity of each setting, effective referral strategies could facilitate service provision to meet the needs and enhance health systems responsiveness at both community and facility levels.

Second, given the high proportion of patient costs out of total societal cost and household income, social financing schemes (e.g. voucher system, conditional cash transfer or community-based financing) should be encouraged for poor households, to reduce potentially catastrophic health expenditures that can result from receiving care especially at child delivery [25]. While the patient costs at community level may seem negligible, service fees and transportation costs are major cost drivers at the facility level. Such cost barriers may be prohibitive to individuals with fewer resources, who may also be at the highest risk of adverse outcomes [26]. Through demand side financing support, ANC services could be more person-centered and cost-effective by reducing waiting time and transportation costs, and improving service quality based on individual pregnancy stage and barriers in care seeking.

### Limitations

With regards to the field data collection for service content and costs, our sampling of the facilities and pregnant women were purposive based on their availability on any pre-scheduled date that ANC service provision operates. We could not address the confounding effects of other potential factors that may affect different care seeking choice at a pregnancy stage. Since our data collection was conducted in rural Gaibandha district, the results might not necessarily reflect service practices and costs in other districts or urban settings. We did not include capital costs or other overhead costs in the cost calculation and hence may underestimate the service provision cost. Measuring accurate marginal costs of service provision in a rural community was challenging due to the unpredictable availability of drugs and staff. This study estimated drug costs based on reported availability, not actual stock level. Considering frequent supply shortage, our provider and patient costs may be underestimated in case patients had to purchase drugs from other private pharmacy stores. Measuring wage loss for indirect patient costs was also a challenge given the high level of informal workers with substantial variation of and unsystematic information about their income. Future studies may increase sample size and collect samples multiple times in different seasons to reduce the effect of seasonality and other potential selection biases. Despite the limitations, this study addressed several complex aspects related to measurement of ANC contents and costs including both health systems and patients’ perspectives in resource-limited settings.

## Conclusion

The concept of effective coverage emphasizes the fact that health impact can be achieved only if health care services are delivered at adequate quality [27]. The study reveals that stage of pregnancy in care-seeking, availability of antenatal care (ANC) subcomponents and provider and patient costs differ by service provision setting, resulting in different quality and cost profiles of ANC available to residents of rural Gaibandha district. Early pregnancy identification and referral coordination between community and facility level with appropriate local incentives could not only promote care-seeking but also enhance the function of primary health systems in rural Bangladesh, where the health system consists of various service provision channels and levels (e.g. informal providers, NGOs, public and private clinics) [28]. Addressing both supply-side and demand-side constraints to the optimal provision of quality care is necessary to shift the needle on adverse pregnancy outcomes currently documented at high rates in these rural, remote communities.

## Supplementary information

**Additional file 1: Table S1.** Percentage distribution of antenatal care service contents in community and facility levels.

## Data Availability

The datasets collected and analyzed during the current study are available from the corresponding author on reasonable request.

## Abbreviations

ANC: Antenatal Care
CC: Community Clinic
CHW: Community Health Worker
DFID: Department for International Development
EMoC: Emergency Obstetric Care
FPI: Family Planning Inspector
FWA: Family Welfare Assistant
FWC: Family Welfare Center
FWV: Family Welfare Visitor
GA: Gestational Age
MBBS: Bachelor of Medicine, Bachelor of Surgery
MCWC: Maternal and Child Welfare Center
NGO: Non-Governmental Organization
QSDS: Quantitative Service Delivery Survey
SACMO: Sub-Assistant Community Medical Officer
SARA: Service Availability and Readiness Assessment
SK: Shyastho Kormi
SPA: Service Provision Assessment
UHC: Upazila Health Complex
USAID: United States Agency for International Development
WHO: World Health Organization
