# Supplementary material for: Antenatal care in rural Bangladesh: current state of costs, content and recommendations for effective service delivery
Source: BMC Health Serv Res. 2019 Nov 21;19:861. doi: 10.1186/s12913-019-4696-7 (PMC6869180; doi:10.1186/s12913-019-4696-7)
Supplement: Supplementary file 1 — Additional file 1: Table S1. Percentage distribution of antenatal care service contents in community and facility levels. [file 12913_2019_4696_MOESM1_ESM.docx]

**Table S1. Percentage distribution of antenatal care service contents in community and facility levels**

|  | Community | Facility |  | Community | | Facility | |
| --- | --- | --- | --- | --- | --- | --- | --- |
|  | (n=34) | (n=36) |  | n | % | n | % |
| 1. Client History (first visit and not identified | 20 | 28 | Client’s age | 20 | 100% | 23 | 82% |
|  |  |  | Medications the client is taking | 7 | 35% | 6 | 21% |
|  |  |  | Date client’s last menstrual period began | 20 | 100% | 26 | 93% |
|  |  |  | Number of prior pregnancies client has had | 20 | 100% | 26 | 93% |
| 2. Aspects of Prior Pregnancies--(Not first pregnancy/not identified, | 20 | 22 | Prior stillbirth(s) | 4 | 20% | 7 | 32% |
|  |  |  | Infant(s) who died in the first month of life | 4 | 20% | 6 | 27% |
|  |  |  | Heavy bleeding, during or after delivery | 1 | 5% | 0 | 0% |
|  |  |  | Previous assisted delivery (c-section, ventouse, or forceps) | 3 | 15% | 9 | 41% |
|  |  |  | Previous spontaneous abortions | 3 | 15% | 1 | 5% |
|  |  |  | Previous menstrual regulation and induced abortion | 2 | 10% | 1 | 5% |
|  |  |  | Previous multiple pregnancies | 2 | 10% | 1 | 5% |
|  |  |  | Previous prolonged labor | 1 | 5% | 1 | 5% |
|  |  |  | Previous pregnancy-induced hypertension | 0 | 0% | 0 | 0% |
|  |  |  | Previous pregnancy-related convulsions | 0 | 0% | 0 | 0% |
|  |  |  | High fever or infection during prior pregnancy/pregnancies | 0 | 0% | 0 | 0% |
| 3. Danger Signs of Current Pregnancy | 34 | 36 | Vomitting,/aversion of food | 10 | 29% | 21 | 58% |
|  |  |  | Vaginal bleeding | 6 | 18% | 2 | 6% |
|  |  |  | Foul smelling discharge | 5 | 15% | 5 | 14% |
|  |  |  | Fever | 7 | 21% | 4 | 11% |
|  |  |  | Headache or blurred vision | 8 | 24% | 3 | 8% |
|  |  |  | Swollen face, hands or legs | 8 | 24% | 3 | 8% |
|  |  |  | Severe abdominal pain | 4 | 12% | 7 | 19% |
|  |  |  | Tiredness or breathlessness | 1 | 3% | 4 | 11% |
|  |  |  | Decreased or absent fetal movement | 3 | 9% | 9 | 25% |
|  |  |  | Persistent cough or difficulty breathing for 3 weeks or longer | 1 | 3% | 0 | 0% |
|  |  |  | Convulsions (pre-eclampsia/eclampsia) | 7 | 21% | 2 | 6% |
|  |  |  | Frequent or painful urination | 3 | 9% | 2 | 6% |
| 4. Physical Examination | 34 | 36 | Take the client’s blood pressure | 34 | 100% | 31 | 86% |
|  |  |  | Weight/height | 34 | 100% | 35 | 97% |
|  |  |  | Conjunctiva/palms for anemia | 9 | 26% | 15 | 42% |
|  |  |  | Legs/feet/hands for edema | 10 | 29% | 4 | 11% |
|  |  |  | Fetal presentation | 12 | 35% | 23 | 64% |
|  |  |  | Uterine height | 13 | 38% | 21 | 58% |
|  |  |  | Fetal heartbeat (after 28 weeks) | 9 | 26% | 0 | 0% |
|  |  |  | Swollen glands | 0 | 0% | 1 | 3% |
|  |  |  | Ultrasound/refer client for ultrasound | 7 | 21% | 9 | 25% |
|  |  |  | Vaginal examination/exam of perineal area | 2 | 6% | 1 | 3% |
|  |  |  | Breasts | 0 | 0% | 0 | 0% |
| 5. Routine Tests | 34 | 36 | Anemia test (asked/performed/referred) | 11 | 32% | 3 | 8% |
|  |  |  | Blood grouping (asked/performed/referred) | 10 | 29% | 8 | 22% |
|  |  |  | Any urine test (asked/performed/referred) | 11 | 32% | 6 | 17% |
|  |  |  | VDRL (Syphillis etc.) test (asked/performed/referred) | 0 | 0% | 3 | 8% |
| 6. Maintaining a Healthy Pregnancy | 34 | 36 | Discussed nutrition during the pregnancy | 22 | 65% | 14 | 39% |
|  |  |  | Informed the client about the progress of the pregnancy | 11 | 32% | 22 | 61% |
|  |  |  | Discussed the importance of at least 4 ANC visits | 6 | 18% | 2 | 6% |
|  |  |  | Informed or gave a card on next ANC scheduled visits | 30 | 88% | 26 | 72% |
| 7. Iron Prophylaxis | 34 | 36 | Prescribed or gave iron pills or folic acid (IFA) | 34 | 100% | 35 | 97% |
|  |  |  | Explained the purpose of iron or folic acid | 6 | 18% | 3 | 8% |
|  |  |  | Explained how to take iron or folic-acid pills | 25 | 74% | 20 | 56% |
|  |  |  | Explained side effects of iron pills | 3 | 9% | 0 | 0% |
| 8. Tetanus Toxoid Injection (GA >13 weeks) | 26 | 34 | Prescribed or gave a tetanus toxoid (TT) injection | 16 | 62% | 17 | 50% |
|  | 26 | 34 | Explained the purpose of the TT injection | 0 | 0% | 1 | 3% |
| 9. Deworming | 34 | 36 | Prescribed or gave Mebendazole/ Albendazole | 0 | 0% | 0 | 0% |
|  |  |  | Explained the purpose of Mebendazole/ Albendazole | 0 | 0% | 0 | 0% |
| 10. Preparation for Delivery (GA >27 week, | 20 | 15 | Asked the client where she will deliver | 8 | 40% | 4 | 27% |
|  |  |  | Advised to prepare for delivery (e.g. money, emergency transportation) | 9 | 45% | 3 | 20% |
|  |  |  | Advised to use a skilled health worker for delivery | 0 | 0% | 1 | 7% |
|  |  |  | Discussed what items to have on hand at home for emergencies (e.g. Birth kit) | 0 | 0% | 0 | 0% |
| 11.Newborn and Postpartum Recommendations (GA > 27 week, | 20 | 15 | Newborn care (i.e. warmth, hygiene and cord care) | 0 | 0% | 0 | 0% |
|  |  |  | Early initiation and prolonged breastfeeding | 0 | 0% | 1 | 7% |
|  |  |  | Exclusive breastfeeding (e.g. 6 months) | 0 | 0% | 1 | 7% |
|  |  |  | Importance of vaccination for the newborn | 0 | 0% | 0 | 0% |
|  |  |  | Family planning options for after delivery | 0 | 0% | 0 | 0% |
